# Light-Triggered Graphene/Black Phosphorus Heterostructure FET Platform for Ultrasensitive Detection of Alzheimer’s Disease Biomarkers at the Zeptomole Level

**DOI:** 10.34133/research.0772

**Published:** 2025-08-14

**Authors:** Huide Wang, Meng Qiu, Chen Wang, Liding Zhang, Ning Fan, Zhi Chen, Yi Liu, Tianzhong Li, Ziqian Wang, Yihan Zhu, Yule Zhang, Xilin Tian, Yun Wang, Mingmin Yang, Dianyuan Fan, Qingming Luo, Ke Jiang, Haiming Luo, Han Zhang

**Affiliations:** ^1^ State Key Laboratory of Radio frequency Heterogeneous integration, International Collaborative Laboratory of 2D Materials for Optoelectronics Science and Technology, Institute for Advanced Study in Nuclear Energy & Safety, Interdisciplinary Center of High Magnetic Field Physics of Shenzhen University, College of Physics and Optoelectronic Engineering, Shenzhen University, Shenzhen, China.; ^2^ Key Laboratory of Marine Chemistry Theory and Technology (Ministry of Education), College of Chemistry and Chemical Engineering, Ocean University of China, Qingdao, China.; ^3^ Britton Chance Center for Biomedical Photonics, Wuhan National Laboratory for Optoelectronics, MoE Key Laboratory for Biomedical Photonics, Huazhong University of Science and Technology, Wuhan, China.; ^4^ State Key Laboratory of Digital Medical Engineering, Key Laboratory of Biomedical Engineering of Hainan Province, School of Biomedical Engineering, Hainan University, Haikou, China.; ^5^ Shenzhen Eye Hospital, Shenzhen Eye Medical Center, Southern Medical University, Shenzhen 518040, China.; ^6^ Department of Chemistry, Korea University, Seoul, South Korea.; ^7^ Research Unit of Multimodal Cross Scale Neural Signal Detection and Imaging, Chinese Academy of Medical Sciences, HUST-Suzhou Institute for Brainsmatics, JITRI, Suzhou, China.; ^8^ State Key Laboratory of Luminescence and Applications, Changchun Institute of Optics, Fine Mechanics and Physics, Chinese Academy of Sciences, Changchun, China.; ^9^ Key Laboratory of Organosilicon Chemistry and Material Technology (Ministry of Education), College of Material Chemistry and Chemical Engineering, Hangzhou Normal University, Hangzhou, China.

## Abstract

Due to the low concentration of amyloid-beta (Aβ) in plasma and the high content of interfering factors, the conventional detection method for the quantification of Aβ still faces the problem of insufficient limit of detection (LOD). In this work, we propose a new light-triggered graphene–black phosphorus heterostructure (G-BP) field-effect transistor (FET) biosensing platform that achieves a ​​marked​​ reduction in the LOD. The LOD for Alzheimer’s disease (AD) biomarker Aβ_42_ detection using the G-BP FET is as low as 235.1 zM (2.351 × 10^−19^ M), which is the lowest value reported to date and is approximately 2 to 3 orders of magnitude lower than other reported biosensing platforms. The G-BP FET platform provides precise, real-time guidance for non-invasive early diagnosis, disease monitoring, and personalized treatment plans for AD. Moreover, this method has good scalability and potential applications in other areas, including early detection of cancer and other major chronic diseases.

## Introduction

Alzheimer’s disease (AD) is characterized by progressive neuropathology and brain atrophy, affecting over 50 million individuals, with projections indicating that this number will reach approximately 152 million by 2050 [1,2]. Amyloid-beta (Aβ) plaques and neurofibrillary tangles (NFTs) composed of phosphorylated tau (p-tau) are widely recognized as the hallmark pathological changes of AD. Among these, aggregated Aβ exhibits critical neurotoxic effects, leading to dendritic retraction and neuronal degeneration, ultimately resulting in memory impairment [3–7]. The primary Aβ subtypes are Aβ_40_ and Aβ_42_, with Aβ_42_ being more prone to fibrillization and demonstrating a stronger tendency for aggregation [2]. The effectiveness of AD treatment depends on how early the treatment is started [8,9]. Therefore, there is an urgent need to develop screening methods that can accurately detect the early stages of AD. In recent decades, biomarker-based non-imaging diagnostic methods utilizing cerebrospinal fluid (CSF), including techniques such as enzyme-linked immunosorbent assay [10,11], nanoparticle-based immunoassays [12], surface-enhanced Raman spectroscopy [13,14], fluorescence biosensors [15], and electrochemical biosensors [16–18], have promoted the development of early diagnosis technology for brain diseases. Recently, minimally invasive detection techniques based on blood biomarkers have received attention, due to better patient compliance and ease for clinical translation. However, the complexity of substances and the presence of numerous interfering signals in blood, coupled with the blood–brain barrier that leads to markedly​ lower concentrations of biomarkers such as Aβ in blood compared to CSF, pose challenges for traditional detection methods when applied to the detection of blood-based biomarkers. For instance, the limit of detection (LOD) and specificity may not meet the necessary requirements [19,20]. Despite these limitations, plasma Aβ_42_ levels and the Aβ_42_/Aβ_40_ ratio have demonstrated ​​substantial clinical relevance, correlating with cerebral amyloid deposition and serving as predictive indicators of cognitive decline in cohort studies [21–23]. Critically, blood-based Aβ_42_ assays achieve reliable detection during the preclinical and mild cognitive impairment stages of AD, enabling timely intervention before symptom onset [24]. Furthermore, most of the Aβ_42_ in blood exists as monomers, making monomer-targeted detection clinically feasible and essential for minimizing false negatives in early screening [25].

Biosensors based on field-effect transistors (FETs) exhibit substantial advantages such as high integration, inherent signal amplification capability, low energy consumption, and ultra-short response times [26]. Caras and Janata [27] pioneered the innovative concept of combining FETs with enzymes, successfully immobilizing penicillinase on a FET with doped silicon as the channel material, achieving efficient detection of penicillin with an LOD of 0.2 mM, a response time of 25 s, and a sensing area of only 0.5 mm^2^. Currently, commercially available detection platforms based on FETs are primarily used for continuous glucose monitoring, among other applications [28,29]. As applications and research progressed, the sensitivity of early FET biosensors became increasingly limited by their channel materials, such as silicon and tin oxide. Compared to 3-dimensional bulk channel materials, 2-dimensional (2D) materials represented by graphene (G) have an ultra-high specific surface area and ultra-thin thickness, which can serve as FET channel materials, achieving extremely high loading capacity and very low signal noise, leading to an extremely low theoretical LOD [7,30], among which the LOD for nucleic acid molecules is as low as 600 zM [31]. G FETs have demonstrated excellent performance in the detection of Aβ_42_. In 2011, Cheng et al. [32] discovered that Aβ_42_ could induce a ​​substantial​ change in the channel current of G FETs, thereby proving the feasibility of using G FETs for the detection of Aβ_42_. Subsequently, researchers confirmed that other materials (such as reduced graphene oxide [33] and carbon nanotubes [34,35]) could also serve as FET channel materials for detecting Aβ_42_, with an LOD as low as 45 aM [35]. By virtue of surface functionalization [36] of the channel material or the use of aptamers [7,35,37] corresponding to Aβ_42_ proteins as the target analyte, the limitations imposed by the material’s inherent properties and the Debye length of the detection environment can be overcome. Up to now, femtomolar level of LOD of FET detection for Aβ_42_ can be achieved by these strategies [33]; however, further improving sensitivity to meet clinical standards poses substantial​​ challenges.

Here, we report a graphene–black phosphorus heterojunction (G-BP) FET optoelectronic biosensing platform for the precise and sensitive detection of AD biomarkers in blood. In the as-designed heterostructure, G serves as the channel material and BP, with its excellent photoresponse properties, serves as the photoactive layer. Under dark conditions, due to the high quality of the prepared G-BP FET and proper control of the Debye length, the Aβ_42_ as a gate voltage signal increases the G channel current, thereby achieving effective detection of Aβ_42_ with an LOD as low as 2.767 × 10^−18^ M (which is equivalent to 12.49 ag/ml or ∼66.6 Aβ_42_ molecules in 40 μl of detection solution). Under illumination, the same concentration of Aβ_42_ not only increases the G channel current but also intensifies the band bending of BP. The photogenerated electron–hole pairs in BP are further separated compared to before the addition of Aβ_42_, resulting in fewer photogenerated holes remaining in BP and a stronger photogating effect corresponding to the photogenerated electrons in BP, which further increases the G channel current. Therefore, the current change in the G-BP FET caused by the same concentration of Aβ_42_ under illumination is markedly greater than that under dark conditions, which means that the LOD under illumination is much lower, reaching 2.351 × 10^−19^ M (which is equivalent to 1.061 ag/ml or ∼5.7 Aβ_42_ molecules in 40 μl of detection solution). Compared to the biosensing platforms reported so far, the LOD we achieved for the detection of Aβ_42_ is the lowest, 2 to 3 orders of magnitude lower than other biosensing platforms. Due to the near-instantaneous nature of light triggering, the detection time under illumination is almost as short as that under dark conditions, approximately 5 min. With the help of this G-BP FET optoelectronic biosensing platform, ultra-sensitive detection of these AD biomarkers in blood can be achieved within several minutes. These results indicate that the light-triggered G-BP FET biosensor has great potential in early diagnosis and large-scale screening tests of AD.

## Results and Discussion

### Schematic diagram of the light-triggered G-BP FET biosensor

**Fig. 1.**
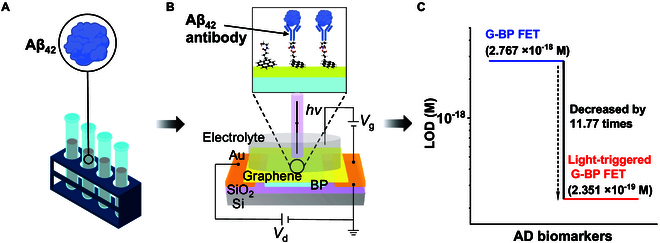
Schematic diagram of G-BP FET biosensor operation procedure. (A) Schematic diagram of the Aβ_42_ antigen. (B) Structural and principle diagram of light-triggered G-BP FET. (C) Effect of light on the detection of LOD of Aβ_42_ protein by the G-BP FET biosensor. In the dark state, the LOD of the G-BP FET biosensor is 2.767 aM. In the light state, the LOD of the G-BP FET biosensor is 235.1 zM.

AD progression involves multiple biomarkers, such as Aβ_40_, T-tau, P-tau, plasma neurofilament light chain, and plasma glial fibrillary acidic protein. Among these, Aβ_42_ is one of the most extensively studied AD biomarkers due to its critical neurotoxic effects in synaptic degeneration. To validate the proof of concept of our light-triggered G-BP FET biosensing platform, we selected Aβ_42_ as a representative target analyte to demonstrate its ultra-sensitive detection capability [38]. A schematic illustration of the Aβ_42_ antigen protein is shown in Fig. 1A. The preparation method for Aβ_42_, as well as Aβ_38_ and Aβ_40_ used in subsequent experiments, refers to our previous report [39]. The device structure is depicted in Fig. 1B, where G serves as the sensing channel of the device, and BP beneath the G absorbs light and generates a photogating effect to further amplify the sensing signal from the G. The detection process encompasses the modification of G surfaces with 1-pyrenebutyric acid N-hydroxysuccinimide ester (PBASE), the conjugation of Aβ_42_ antibodies, the passivation of excess PBASE, and the collection of sensing current signals generated upon the specific binding of Aβ_42_ antigenic proteins to Aβ_42_ antibodies. Due to the high specificity of the Aβ_42_ antibodies employed, G-BP FET modified with Aβ_42_ antibodies exhibits superior detection performance. The LOD of the G-BP FET for Aβ_42_ antigenic proteins is as low as 2.767 × 10^−18^ M, which is markedly​​ lower than the results reported in other studies. More importantly, by subjecting the device to illumination, the signal current of the device is further amplified due to the grating effect of photogenerated electrons in BP, allowing the LOD of the G-BP FET to be reduced to 2.351 × 10^−19^ M (Fig. 1C).

### Construction and characterization of biosensor

BP and G were used to prepare the G-BP heterojunction, and Raman spectroscopy showed that the G-BP heterojunction was successfully constructed (Fig. S1). The G-BP heterojunction is used as a channel material to fabricate FET devices (Figs. S2 and S3). In this system, G has 3 main functions. First, due to its high carrier mobility, G can act as a conductive channel [40]. Second, G’s large specific surface area allows it to act as a sensing interface [41]. The modification of biomolecules on the G surface generates electrostatic gating that regulates channel conductivity, enabling efficient biomolecular recognition. Finally, G encapsulates BP, thereby isolating it from air and water and acting as a passivating agent [42]. As shown in Fig. S4, the Raman spectra of G-BP tested over 20 days of ambient exposure exhibited no variation in characteristic peaks, with stable peak intensities, strongly demonstrating the feasibility of G-encapsulated BP. BP has excellent optical properties and is used as a photosensitive material [43]. The G-BP FET biosensor platform developed in this study is shown in Fig. S5. FET is fixed to the chip carrier, far above the biosensing channel of polydimethylsiloxane (PDMS) used to contain the electrolyte solution and the liquid to be detected. Figure S6 depicts an optical image of a G-BP FET sensor with a sensing area size of 100 × 40 μm^2^ (L × W).

**Fig. 2.**
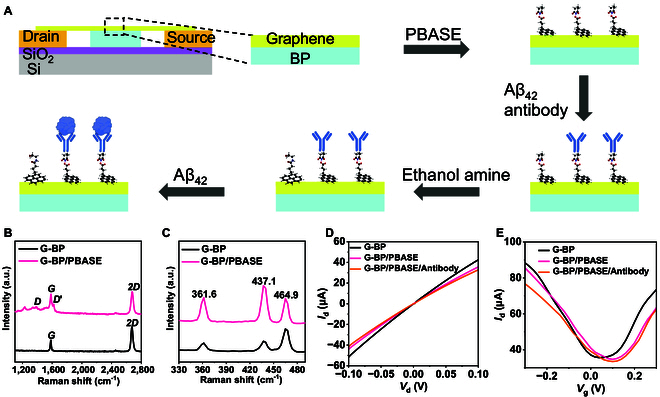
Construction and characterization of the G-BP FET biosensor. (A) Schematic diagram of G-BP FET functionalization and detection of Aβ_42_ protein. (B) Raman spectra (1,100 to 2,800 cm^−1^) of G-BP before and after modification of PBASE. (C) Raman spectra (330 to 490 cm^−1^) of G-BP before and after modification of PBASE. (D) Output characteristic curve of G-BP FET during functionalization (PBASE and antibody modifications). (E) Transfer characteristic curve of G-BP FET during functionalization (*V*_d_ = 0.1 V).

The functionalization of sensing surfaces is an important step in the construction of biosensors. Figure 2A illustrates in detail the process of functionalization of G-BP FET and detection of Aβ_42_ protein. The PBASE, a commonly used and effective interfacial coupling agent, is utilized to immobilize Aβ_42_ antibodies onto the G surface. The pyrene group at one end of PBASE is π–π stacked with G to form a self-assembled layer, which is uniformly modified on the surface of G [44]. The N-hydroxysuccinimide ester at the other end can react with the amine group on the modified Aβ_42_ antibody through a primary amine reaction, allowing the antibody to be fixed on the sensing surface [45]. In order to verify the successful functionalization of PBASE, Raman spectroscopy and device electrical performance data were obtained. Figure 2B and C show the Raman spectra of different bands before and after modifying PBASE. The 3 characteristic peaks in Fig. 2B represent BP [46]. As shown in Fig. 2C, the peaks labeled *G* and *2D* are characteristic peaks of G [47]. The *G* peak is related to the lattice vibration mode, and the *2D* peak is derived from second-order Raman scattering. After modification with PBASE on the G surface, the characteristic peak exhibits a blue shift, indicating that the modification with PBASE has led to p-doping of the device, a doping effect resulting from the π–π interactions between PBASE and G [48]. Additionally, the average intensity ratio of the *2D* and *G* peaks (*I*_2D_/*I*_G_) serves as an indicator of the interaction between PBASE and G. Owing to the existence of some disorder on the G [45], the *I*_2D_/*I*_G_ ratio of G modified with PBASE (*I*_2D_/*I*_G_ = 1.19) was significantly lower than that of G unmodified with PBASE (*I*_2D_/*I*_G_ = 1.76) [7].

In order to assess the degree of functionalization of the G surface, we performed electrical performance tests on the devices. Figure 2D shows the output characteristic curve in the range of −0.1 to +0.1 V during device functionalization. After functionalization of PBASE and fixation of Aβ_42_ antibodies to the G surface, the slope of the curve gradually decreased (d*I*_d_/d*V*_d_), and this slope difference indicated the successful introduction of Aβ_42_ antibodies. Subsequently, the transfer characteristic curves of G-BP FET devices after each modification process were tested (Fig. 2E). The G-BP FET device exhibits ambipolar characteristics with p-type dominance. After the modification of PBASE, a positive shift of the transfer characteristic curve was observed due to the p-doping effect of the pyrene group. Subsequently, the transfer curve after fixing the antibody was negatively shifted, which was due to the positive charge of the antibody in the detection environment, and the n-doping effect on the G. The change of output curve of the G-BP FET biosensor under different *V*_g_ values is shown in Fig. S7. *V*_g_ ranges from 0 to 0.4 V, and the step size is 0.1 V. When *V*_d_ is held constant, an increase in *V*_g_ leads to an increase in *I*_d_, a phenomenon consistent with the expected characteristics of an n-type semiconductor. Furthermore, the linearity of the output characteristic curve indicates that the device has a highly stable ohmic contact, thereby suggesting that the G-BP FET biosensor provides a reliable electrical signal for the detection of Aβ_42_ antigen.

### Electrostatic photogating effect

**Fig. 3.**
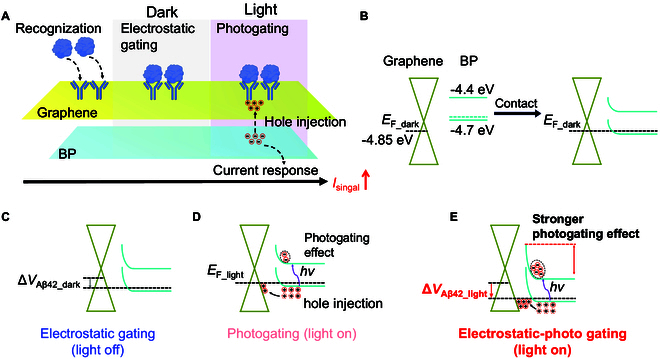
Mechanism of electrostatic photogating. (A) Schematic illustration of Aβ_42_ protein detection by G-BP FET using the electrostatic photogating mechanism. (B) Energy band changes before and after the formation of heterojunction between G and BP. (C) Energy band variation diagram of G-BP in the electrostatic gating process. (D) Energy band variation diagram of G-BP in the photogating process. (E) Energy band variation diagram of G-BP in the electrostatic photogating process.

Figure 3A is a schematic diagram of the detection process of Aβ_42_ by the G-BP FET biosensing platform. Firstly, before the functionalization of the device, G forms a heterojunction with BP, inducing ​​substantial changes in the energy band, as shown in Fig. 3B. The Fermi levels of G and BP before contact are approximately −4.85 and −4.7 eV, respectively [46]. Upon contact, the formation of a heterojunction between G and BP causes band bending in BP, resulting in the creation of an energy barrier. Figure 3 describes the principle of G-BP FET detection of Aβ_42_ from the energy band perspective. The detection mechanism under dark conditions is shown in the middle of Fig. 3A. Aβ_42_ is negatively charged in our detection solution (pH = 8.5) and physiological conditions [33]. When Aβ_42_ (antigen) binds to the corresponding antibody on the device surface, the negatively charged Aβ_42_ will exert an electrostatic gating effect on the G surface, in which an electric double layer (EDL) forms between Aβ_42_ and the device surface [47–49], as shown in Fig. S8, leading to p-type doping of G, a decrease in the Fermi level by an amount Δ*V*_Aβ42_dark_, and consequently causing the G channel current to increase exponentially corresponding to Δ*V*_Aβ42_dark_ (see Section S1), as depicted in Fig. 3C. That is to say, even an extremely small amount of Aβ_42_ can markedly increase the device’s channel current through electrostatic gating, which supports the ultra-sensitive detection of Aβ_42_, and subsequent experimental results show that under dark conditions, the LOD for Aβ_42_ is as low as 2.767 × 10^−18^ M.

It is noteworthy that in FET biosensors, the gate control capability of the analyte over the channel is constrained by the screening effect of the test solution on the electric field, which substantially attenuates the biosensor signal [50]. Modeling of the screening effect typically relies on a simple capacitive model based on the linearized Poisson–Boltzmann equation, which is used to describe the distribution of potential attenuation in the presence of mobile charges. The Debye length stems from this concept, characterizing the spatial scale of potential decay as a characteristic value, providing a useful standard for the spatial scale of electric field screening [50]. Under physiological conditions, the Debye length is less than 1 nm, whereas most antibodies are about 10 to 15 nm in length. In our previous research, the length of the Aβ_42_ antibody was measured to be approximately 64.9 nm [2,50], indicating that the ideal Debye length in our detection environment needs to be greater than 64.9 nm. Therefore, we increased the Debye length from the physiological level of 1 to ~23 nm by using a low-concentration buffer (0.001× phosphate-buffered saline [PBS]) [51]. Our experimental results indicate that the G-BP FET can already achieve sensitive detection of Aβ_42_ in a 0.001× PBS buffer solution, which seems to be inconsistent with the aforementioned Debye length requirement. We suspect that this anomalous phenomenon may arise from 2 factors: (a) Some Aβ_42_ specifically binding to the antibody surface in a non-perpendicular orientation, which may make the distance between Aβ_42_ and the G surface smaller than the original Debye length; and (b) the reduced Debye volume (the available volume of EDL) caused by surface indentation of G induced by the underlying thicker BP layer and localized wrinkles generated during the G transfer process. The reduction in Debye volume enables the G-BP FET to be used for Aβ_42_ detection beyond the Debye length.

**Fig. 4.**
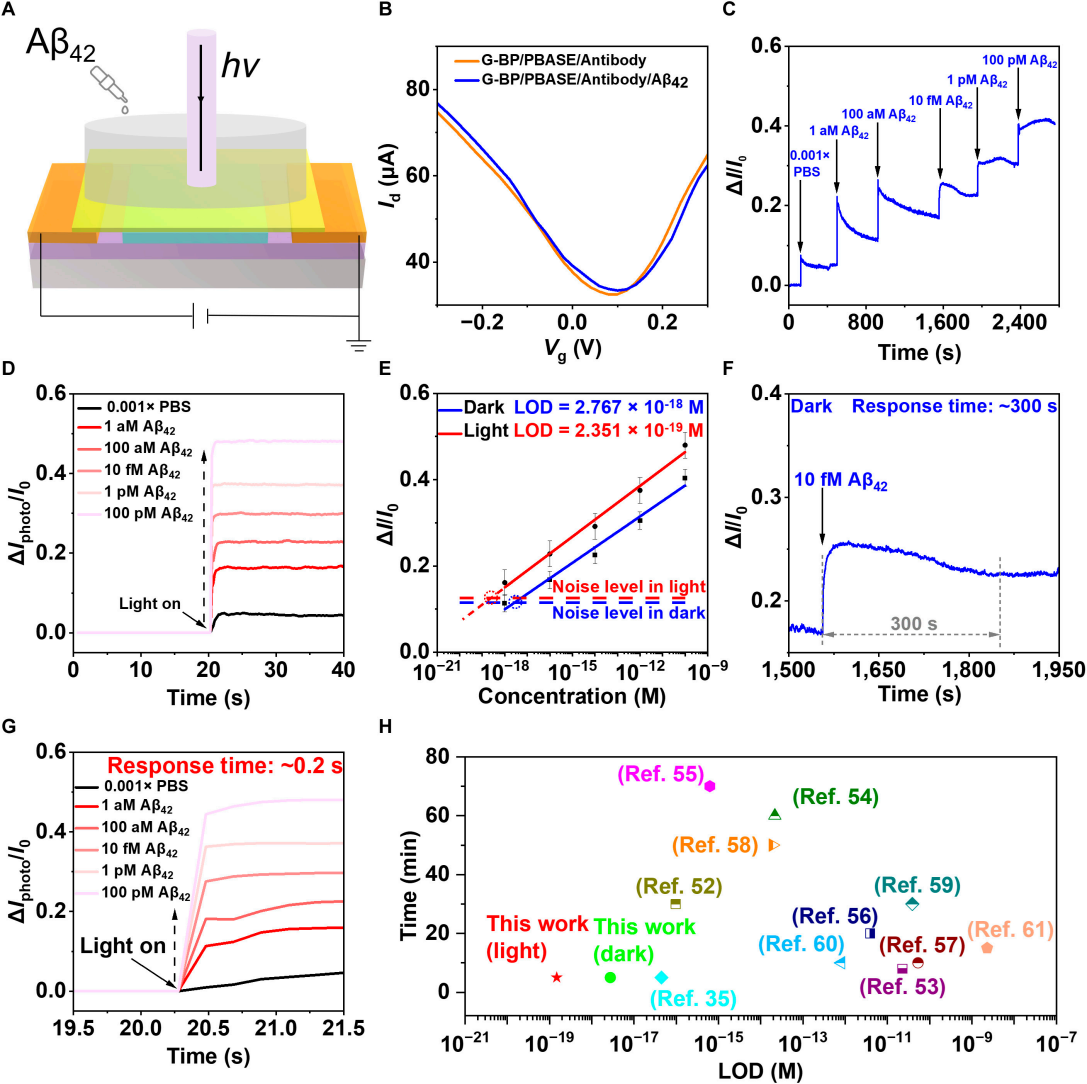
Aβ_42_ biomarker detection. (A) Schematic diagram of detecting Aβ_42_ by G-BP FET. (B) The transfer characteristic curve of Aβ_42_ was detected by G-BP FET (*V*_d_ = 0.1 V). (C) Real-time response of G-BP FET to Aβ_42_ in 0.001× PBS in the dark state. (D) The response curve of the G-BP FET biosensor changed with time after adding Aβ_42_ with a concentration of 0 to 100 pM to 0.001× PBS. From 20 s onwards, the device is exposed to light. (E) Relationship between Aβ_42_ concentration and response signal. The intersection of noise level and curve is LOD. LOD is 2.767 aM in the dark and 235.1 zM in the light. (F) The response time of 10 fM Aβ_42_ was detected in the dark state. (G) Light response time of the G-BP FET sensor under different Aβ_42_ concentrations. (H) The performance of this work was compared with other methods for the detection of Aβ_42_.

The photon energy of the light source is approximately 2.33 eV, exceeding the bandgaps of BP (0.3 eV) and G (0 eV). When photons irradiate the device, valence band electrons in black phosphorus or graphene absorb photon energy and transition to the conduction band, generating photogenerated carriers. When BP was exposed to photons with energy equal to or greater than its bandgap, electrons in the valence band are excited into the conduction band, leaving behind holes. These holes are driven into the G by the built-in electric field, while the photoexcited electrons are trapped in the energy barrier at the BP interface. The accumulated photoexcited electrons act as a local gate, p-doping the G channel, as shown in Fig. 3D, thereby increasing the G channel current. As shown in Fig. S9, the channel current of the pristine G FET hardly changes under illumination, indicating that compared to the G-BP FET, the pristine G FET has a negligible photoresponse. The detection mechanism under light illumination is shown on the right side of Fig. 3A. When Aβ_42_ was added to the detection solution under light illumination, it first causes the G channel current to increase to the same extent as when the same concentration of Aβ_42_ was added in the dark (the Fermi level is lowered by the same degree). This further intensifies the band bending of BP, and the photogenerated electron–hole pairs in BP are more separated than before the addition of the Aβ_42_. The reduction of photogenerated holes remaining in BP leads to a stronger photogating effect corresponding to the photogenerated electrons, which will further increase the G channel current (further lower the Fermi level). Therefore, the change in G-BP FET current caused by the same concentration of Aβ_42_ under illumination (the total change in Fermi level is denoted as Δ*V*_Aβ42_light_) is markedly greater than that under dark conditions, as illustrated in Fig. 3E. This conclusion can also be drawn from the transfer curves in Fig. S10. Specifically, in the dark state, after the addition of 100 pM Aβ_42_ to the G-BP FET detection platform, the Δ*V*_Dirac_ in the transfer curve is 5 mV, and this value ​​markedly​​ increases to 20 mV under illumination. Furthermore, under illumination, the response of the pristine G FET to 100 pM Aβ_42_ is approximately 11.00% (Fig. S11), while the response of the G-BP FET is substantially higher, at 47.70% (Fig. 4D). Based on the analysis above, under illumination, a much lower LOD is achieved, reaching 2.351 × 10^−19^ M.

### Detection of Aβ_42_ protein by light-triggered G-BP FET biosensor

As illustrated in the schematic diagram of the biosensor in Fig. 4A, the G-BP FET biosensor can detect Aβ_42_ by monitoring the changes in surface charge on the channel and channel current. Figure 4B describes the changes of the device’s transfer curves before and after adding Aβ_42_. The specific binding between Aβ_42_ and the corresponding antibody (1F12) caused a positive shift in the transfer curve, indicating that Aβ_42_ introduced a p-doping effect on G.

To evaluate the effectiveness of the G-BP FET biosensor, we meticulously assessed the sensor’s real-time response to Aβ_42_. We defined a parameter ($∆I/I_{0}$) to represent the change in signal, expressed as:

$$∆I/I_{0}=\frac{I-I_{0}}{I_{0}}$$ (1)

where $I_{0}$ and I represent the channel current of the biosensor before and after the introduction of Aβ_42_, respectively.

As shown in Fig. 4C, the response of the biosensor markedly increases with the increasing of concentration of Aβ_42_. The $∆I/I_{0}$ response at Aβ_42_ concentrations of 1 aM and 100 pM is ~11.37% and ~40.10%, respectively. These experimental phenomena confirm that the G-BP FET biosensor exhibits ​​high​ sensing capabilities for Aβ_42_ under dark conditions. Next, we investigated the impact of light on the detection process. Under illumination of 12.7 mW/cm^2^, the detection data for Aβ_42_ by the G-BP FET biosensor is shown in Fig. 4D. The results indicate that Aβ_42_ can also be detected under light conditions, with the response signal being positively correlated with the concentration of Aβ_42_, a trend that is consistent with the dark state. It is worth noting that the biosensor’s response to 100 pM Aβ_42_ is 48.00% and 40.10% under illumination and dark, indicating that light triggering can enhance the biosensing signal.

Additionally, we explored the correlation between the concentration of Aβ_42_ and the response of $∆I/I_{0}$. The error bars were obtained from 3 repeated experiments. Under both conditions, the response of $∆I/I_{0}$ showed a linear relationship with the logarithm of the Aβ_42_ concentration (Fig. 4E), and the linear fits are given by:

$$∆I_{\text{dark}}/I_{0}=0.03588\text{log}C+0.74527$$ (2)

$$∆I_{\text{light}}/I_{0}=0.03920\text{log}C+0.85605$$ (3)

We have thoroughly discussed the origin of the linear relationship between $∆I/I_{0}$ and Aβ_42_ in Section S1. The data indicate that the G-BP FET biosensor exhibits higher sensitivity under illumination (slope = 0.03920) than under dark (slope = 0.03588). Additionally, the LOD can be determined by the intersection of the noise level with the fitting curve (as detailed in Section S2). The results show that the LOD under illumination (235.1 zM) is 11.77 times lower than the LOD under dark (2.767 aM).

The response time is ~300 s under dark, as shown in Fig. 4F extracted from Fig. 4C. Under illumination, the time required for the response signal caused by the photogating effect is only ~0.2 s, as shown in Fig. 4G extracted from Fig. 4D. Therefore, when the G-BP FET is used for detection under illumination, the detection time is ~300.2 s, indicating that the light-triggered G-BP FET almost maintains the fast response characteristic of the G-BP FET used for biosensing.

Furthermore, stability is one of the key indicators for evaluating the sensing performance of the G-BP FET. Therefore, a series of repeatable responses spanning 9 on–off cycles were performed for the G-BP FET sensing platform over a continuous duration of 400 s, with results shown in Fig. S12. The current variation remained largely consistent throughout the 9 light-switching cycles, demonstrating the excellent stability of the system. Next, the optical response performance of 100 pM Aβ_42_ was detected on the 1st and 20th days, respectively, using the same G-BP FET device. The results, as shown in Fig. S13, indicate that the average photocurrent response amplitude remains at 86.04% of the initial value.

We compared with other reported methods from the perspective of LOD and response time [39], as shown in Fig. 4H. The LOD of the light-triggered G-BP FET is about 3 orders of magnitude lower than traditional techniques such as surface-enhanced Raman scattering spectroscopy [14], FET sensors [35,52], electrochemical sensors [53,54], electrochemical immunoluminescence analysis [55], molecular imprinting sensors [56], and other traditional measurement methods [57–61]. Furthermore, the response time of the G-BP FET for biosensing is almost record short.

**Fig. 5.**
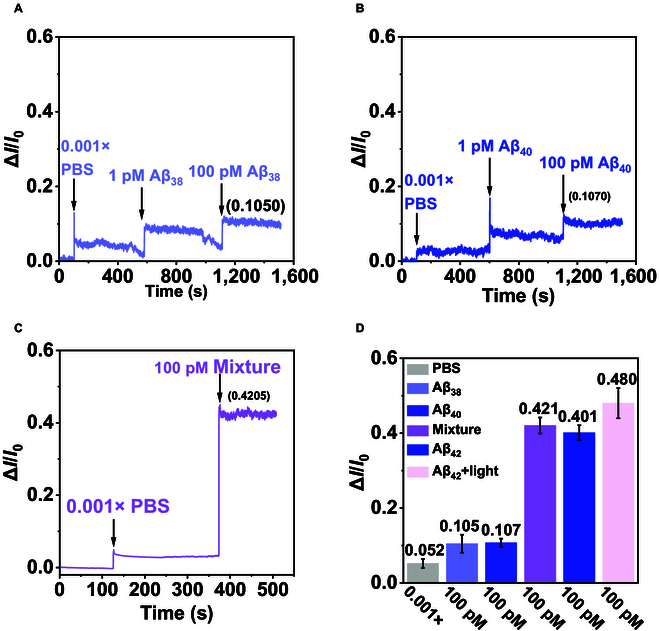
Specificity verification of the G-BP FET biosensor. (A) Real-time response of G-BP FET to Aβ_38_ in 0.001× PBS. (B) Real-time response of G-BP FET to Aβ_40_ in 0.001× PBS. (C) Real-time response of G-BP FET to mixed peptides in 0.001× PBS. (D) Response signals of PBS (0.001×), Aβ_38_ (100 pM), Aβ_40_ (100 pM), mixed peptides (100 pM), and Aβ_42_ (100 pM and 100 pM + light).

We investigated the specificity of the G-BP FET biosensor. Aβ_38_ and Aβ_40_ were introduced for detection under dark, and the results showed that the $∆I/I_{0}$ response of the G-BP FET to 100 pM Aβ_38_ and 100 pM Aβ_40_ were 10.50% and 10.70%, respectively (Fig. 5A and B). Both of these values are lower than the noise caused by pure 0.001× PBS (11.529%), indicating that Aβ_38_ and Aβ_40_ could not be detected. The weak signals exhibited here can be attributed to the non-specific adsorption of Aβ_38_ and Aβ_40_ on the sensing interface. Next, we evaluated the response of the G-BP FET biosensing platform to a mixed peptide solution (containing 100 pM Aβ_38_, Aβ_40_, and Aβ_42_) under dark conditions. As shown in Fig. 5C, the mixed peptides elicited a response signal of approximately 42.10%, demonstrating the platform’s capability to selectively detect Aβ_42_ in complex matrix environments despite the presence of structurally similar Aβ isoforms. Figure 5D summarizes the changes in channel current caused by Aβ_38_, Aβ_40_, and Aβ_42_ at different concentrations, and it can be seen that the G-BP FET responds strongly only to Aβ_42_, while illumination further enhances the response signal, demonstrating the high specificity of the G-BP FET biosensor for Aβ_42_.

To validate the performance of the light-triggered G-BP FET biosensing platform in complex solution environments, the detection capability of the G-BP FET for Aβ_42_ in serum environments was tested. As shown in Fig. S14, the G-BP FET exhibited clear response signals to Aβ_42_ at varying concentrations under both dark and illuminated states, with the response signals increasing markedly as Aβ_42_ concentration rose. This strongly demonstrates the feasibility of the light-triggered G-BP FET biosensor for Aβ_42_ detection in serum environments. By fitting the relationship between response signals and concentrations, a linear fitting function was derived:

$$∆I_{\text{dark}}/I_{0}=0.05346\;\text{log}\;C+1.05967$$ (4)

$$∆I_{\text{light}}/I_{0}=0.05500\;\text{log}\;C+1.12787$$ (5)

Finally, the LOD for Aβ_42_ in serum was calculated, yielding values of 4.440 aM in the dark state and 1.932 aM under illumination.

In addition, the reliance of the G-BP FET sensor on antibodies for target capture introduces inherent limitations to its detection performance, as the sensor’s sensitivity and stability remain constrained by antibody-dependent factors such as binding affinity and degradation resistance, highlighting a current drawback of the platform.

## Conclusion

In summary, we report a light-triggered G-BP FET biosensing platform for precise, sensitive, and fast detection of AD blood-oriented biomarkers. In the as-designed heterojunction, G serves as the channel material, and BP, with its excellent photo responsive performance, acts as the photo driven layer. Under dark, thanks to the high quality of the prepared G-BP FET devices and proper control of the Debye length, effective detection of Aβ_42_ was achieved, with an LOD as low as 2.767 × 10^−18^ M. Under illumination, due to the photogating effect, a much lower LOD was achieved, reaching 1.491 × 10^−19^ M. Among the biosensing platforms reported to date, the LOD we achieved for the detection of Aβ_42_ is the lowest, 3 orders of magnitude lower than other biosensing platforms. The detection time of the light-triggered G-BP FET is ~300.2 s. With the help of this G-BP FET photoelectric sensing platform, ultra-sensitive detection of the AD blood-oriented biomarkers can be achieved within several minutes. These results indicate that the light-triggered G-BP FET biosensor has great potential in the early diagnosis and large-scale screening tests of AD.

## Materials and Methods

### Materials

G was purchased from Nanjing Muke Nanotechnology Co., Ltd. (Nanjing, China). BP was purchased from Nanjing XFNANO Materials Tech Co., Ltd. (Nanjing, China). Aβ_38_, Aβ_40_, and Aβ_42_ were custom-synthesized as lyophilized powders by Royo Biotech Co., Ltd (Shanghai, China) with a purity of >95%. PBASE was acquired from Thermo Fisher Scientific (Waltham, MA).

### Preparation of few-layer black phosphorus

In this study, a mechanical stripping technique was employed to prepare a few-layer BP. First, take a 10-cm-long piece of transparent tape and repeatedly place bulk BP in the middle of the transparent tape, leaving an appropriate amount of BP. Second, fold the tape about 100 times to ensure that the BP is evenly distributed throughout the tape. Then, cut a piece of PDMS film with a size of about 0.5 × 0.5 cm^2^, remove 2 layers of protective film, paste it on a clean glass sheet, paste the folded tape on the PDMS film, apply firm pressure within the specified time, and remove the tape in time. Finally, a thin layer of BP is transferred to the PDMS film, and a small amount of BP layers on the PDMS film are observed using an optical microscope, and a suitable layer is selected for subsequent transfer.

### Preparation of single layer graphene

Monolayer G was obtained by etching copper-based PMMA/G. First, prepare the etching solution. The etching solution consisted of a mixture of 40 ml of 30 g/l hydrogen peroxide and 4 ml of 36% hydrochloric acid. Secondly, cut a piece of copper-based PMMA/G sized approximately 0.25 × 0.25 cm^2^, and immerse it in the etching solution. The etching process was conducted for approximately 10 min. Finally, once the PMMA/G became completely transparent, transfer the clean slide to deionized water for 10 min and repeat the washing process 3 times.

### Fabrication of devices

Initially, transfer the few-layer BP from the PDMS film to the channel of the SiO_2_/Si substrate. Attach the cleaned silicon wafer to the PDMS film, apply gentle pressure, heat to 80 °C, and maintain a constant temperature for 10 min prior to separation. At this stage, a small layer of BP has been successfully transferred to a specific location on the silicon chip. Subsequently, use a silicon wafer with transferred few-layer BP to quickly scoop up the PMMA/G in the deionized water. Then, quickly rinse in isopropyl alcohol. Next, wash the device in acetone for 1 min to eliminate PMMA from the G surface. Lastly, rinse the device 3 times with isopropyl alcohol and dry with nitrogen. Dry the prepared devices overnight under vacuum conditions.

### Functionalization of graphene surface

The prepared devices were soaked in a 5 mM PBASE solution in methanol for 30 min at room temperature, subsequently washed 3 times with 10 μM PBS buffer and deionized water, and then incubated in a 1 μM Aβ_42_ antibody [2,62] solution for 4 h (Fig. S15).

### Characterization

The preparation and functionalization of the materials were characterized by Raman spectroscopy. Raman scattering analysis was conducted using the alpha300 R confocal Raman imaging microscope system (Oxford Instruments WITec). This microscope is equipped with a 532-nm laser serving as the excitation source.

### Device measurement

As a biosensor, PDMS well is positioned above the channel to contain a 40-μl analyte solution, with the gate probe of the probe station serving as the liquid gate electrode. Electrical measurements are conducted at room temperature using a semiconductor analyzer (Keithley Instruments, 4200-SCS). When *V*_g_ = 0 V, the *I*_d_–*V*_d_ output curve is obtained by scanning the *I*_d_ for *V*_d_ ranging from −1 to 1 V. At *V*_d_ = 0.1 V, the *I*_d_–*V*_g_ transfer curve is obtained by scanning the *V*_g_ within the range of −1 to 1 V, and the Dirac point is determined at the juncture where *I*_d_ reaches its minimum value. In the photocurrent test, utilize an optical chopper (OEABT, OBS-C1) to control the ON–OFF state of lighting, with measurements of the light-triggered chemo-transistor conducted in a dark sealed box. The light source is provided by the 532-nm laser.

### Detection of Aβ_42_ protein

The detection of Aβ_42_ was assessed using a semiconductor analyzer and a probe station. The molecular weight of Aβ_42_ used in this work is 4,514.03 g/mol. During measurements in 10 μM PBS, a stable bias (*V*_d_ = 0.1V) is applied between the drain and source. Once the *I*_d_ was stabilized, varying concentrations of Aβ_42_ were introduced for testing. The electrical response signal detected is normalized to Δ*I*/*I*_0_.

## Data Availability

All data relevant to this study are available in the manuscript and supporting information. Raw data are available from the corresponding authors upon reasonable request.

## Supplementary Materials

Supplementary 1 Supplementary Text Figs. S1 to S15 References [63–68]
