# Supplementary material for: Light-Triggered Graphene/Black Phosphorus Heterostructure FET Platform for Ultrasensitive Detection of Alzheimer’s Disease Biomarkers at the Zeptomole Level
Source: Research (Wash D C). 2025 Aug 14;8:0772. doi: 10.34133/research.0772 (PMC12352878; doi:10.34133/research.0772)
Supplement: Supplementary 1 — Supplementary Text Figs. S1 to S15 References [63–68] [file research.0772.f1.docx]

Supplemental Materials

**Light-triggered Graphene/Black Phosphorus Heterostructure FET Platform for Ultrasensitive Detection of Alzheimer's Disease Biomarkers at Zeptomole Level**

Huide Wang^1,^ †, Meng Qiu^2,^ †, Chen Wang^2,^ †, Liding Zhang^3,4^, Ning Fan^5^, Zhi Chen^1,9^, Yi Liu^1^, Tianzhong Li^1^, Ziqian Wang^1^, Yihan Zhu^1^, Yule Zhang^1^, Xilin Tian^1^, Yun Wang^5^, Mingmin Yang^5^, Dianyuan Fan^1^, Qingming Luo^4,6^, Ke Jiang^7^, Haiming Luo^3,4,6^*, Han Zhang^1,8^*

**Table of contents**

**Supplementary Text**

Section S1. Connection between the concentration of Aβ_42_ and the signal response.

Section S2. Calculation method of limit of detection (LOD).

**Supplementary Figures**

Figure S1. Raman spectra of G, BP and G-BP heterojunctions.

Figure S2. Schematic diagram of the G-BP FET construction process.

Figure S3. Construct optical image of G-BP FET.

Figure S4. Raman spectral evolution of G-encapsulated BP under ambient exposure for 20 days.

Figure S5. Physical diagram of G-BP FET.

Figure S6. Optical image of G-BP FET.

Figure S7. Output characteristic curve of G-BP FET.

Figure S8. Schematic diagram of EDL.

Figure S9. Effect of light on the channel current of pristine graphene FET.

Figure S10. Transfer characteristic curves of G-BP FET under different test conditions.

Figure S11. Response of pristine graphene FET to 100pM Aβ_42_.

Figure S12. Stability of G-BP FET assay system with on-off illumination for 9 times.

Figure S13. The light response curves of 100 pM Aβ_42_ were detected by the same G-BP FET biosensor on the 1st and 20th days.

Figure S14. Detection of Aβ_42_ biomarkers in serum.

Figure S15. The variation trend of drain current over time during the antibody modification process.

**Supplementary Text**

**Section S1.** Connection between the concentration of Aβ_42_ and the signal response

Initially, the correlation between the signal response and the concentration of Aβ_42_ in the absence of illumination is inferred. Nernst's equation [39,63,64] delineates the association between the potential of the G-BP FET channel and the concentration of Aβ_42_, expressed as:

$V_{channel}=V_{0}+\frac{RT}{zF}\ln\frac{C_{protein}}{C^{\theta}}$ (1)

where additional parameters include: $V_{channel}$ as the potential of channel, $V_{0}$ as the initial potential, $R$ as the molar gas constant, $T$ as the temperature, $z$ as the charge of Aβ_42_, $F$ as the Faraday constant, $C_{protein}$ as the concentration of Aβ_42_, and $C^{\theta}$ as the standard concentration.

For a BP-FET, carrier mobility can be expressed as [65,66]:

$\mu=\frac{1}{c_{g}}\frac{L}{W}\frac{1}{V_{d}}\frac{dI_{d}}{dV_{g}}$ (2)

Here, the parameters are defined as follows: $\mu$ represents the carrier mobility, $c_{g}$ denotes the gate capacitance, $L$ signifies the channel length, $W$ stands for the channel width, $V_{d}$ indicates the voltage between the source and drain, $I_{d}$ represents the channel current, and $V_{g}$ is the gate voltage.

In the dark state, integrate equation (2) to obtain:

${\Delta I}_{dark}= \mu\frac{W}{L}c_{g}V_{d}(V_{g}-V_{channel})$ (3)

where $I_{dark}$ is the channel current in dark.

Substituting equation (1) into equation (3) yields the relationship between ${\Delta I}_{dark}$ and $C_{protein}$ as:

${\Delta I}_{dark}= \mu\frac{W}{L}c_{g}V_{d}(V_{g}-V_{0}-\frac{RT}{zF}\ln\frac{C_{protein}}{C^{\theta}})$ (4)

where the physical quantities are regarded as constants except ${\Delta I}_{dark}$ and $C_{protein}$. Hence, equation (4) is simplified as:

${\Delta I}_{dark}= A_{dark}\ln C_{protein}+B_{dark}$ (5)

where $A_{dark}$ and $B_{dark}$ are constants. As such, ${\Delta I}_{dark}$ is linearly associated with $\ln C_{protein}$. Consequently, under dark conditions, ${\Delta I}/{I_{0}}$ exhibits a linear relationship with the logarithmic concentration of Aβ_42_ [67].

When illuminated, the channel current is represented by:

${\Delta I}_{light}= \mu\frac{W}{L}c_{g}V_{d}{\Delta V}_{g}$ (6)

During this period, the modulation of photocurrent is attributed to the combined influence of the chemical gating and the photogating. Consequently, ${\Delta V}_{g}$ is decomposed into ${\Delta V}_{g1}$ and ${\Delta V}_{g2}$, where $V_{g1}$ represents the variation in $V_{channel}$ induced by chemical doping, while ${\Delta V}_{g2}$ arises from the localized potential produced by the grating, denoted by ${\Delta V}_{g-photogating}$.

As such, the upon analyte can be ${\Delta I}_{light}$ calculated by:

${\Delta I}_{light}= \mu\frac{W}{L}c_{g}V_{d}({\Delta V}_{channel}+{\Delta V}_{g-photogating})$ (7)

With optical power held constant, alterations in the energy barrier resulting from chemical doping affect ${\Delta V}_{g-photogating}$. Evidently, ${\Delta V}_{g-photogating}$ correlates with $C_{protein}$. Hence, we establish ${\Delta V}_{g-photogating}$=$\beta{\Delta V}_{channel}$. Consequently, ${\Delta I}_{light}$ is represented as:

${\Delta I}_{light}= \mu\frac{W}{L}c_{g}V_{d}(1+\beta){\Delta V}_{channel}$ (8)

Substituting equation (1) into equation (8) yields the relationship between ${\Delta I}_{light}$ and $C_{protein}$ as:

${\Delta I}_{light}= \mu\frac{W}{L}c_{g}V_{d}(1+\beta)(V_{g}-V_{0}-\frac{RT}{zF}\ln\frac{C_{protein}}{C^{\theta}})$ (9)

where the physical quantities are regarded as constants except ${\Delta I}_{light}$ and $C_{protein}$. Hence, equation (9) is simplified as:

${\Delta I}_{light}= A_{light}\ln C_{protein}+B_{light}$ (10)

where $A_{light}$ and $B_{light}$ are constants. As such, ${\Delta I}_{light}$ is linearly associated with $\ln C_{protein}$. Consequently, under light conditions, ${\Delta I}/{I_{0}}$ exhibits a linear relationship with the logarithmic concentration of Aβ_42_.

**Section S2.** Calculation method of limit of detection (LOD)

Prior to calculating LOD, the noise level must be ascertained. We define the noise level as three times the response of the G-BP FET to pure PBS (0.001X; pH=8.5) [68]. The response of 0.001X PBS buffer in dark state was 3.843% and the noise level was 11.529%. The response of 0.001X PBS buffer in light state was 4.193% and the noise level was 12.580%. The intersection of the transistor's linear fitting curve and noise curve is then used to calculate the LOD. LOD was determined to be 2.767 aM (electrostatic gating) and 235.1 zM (electrostatic-photo gating).

**Supplementary Figures**

**Figure S1.** Raman spectra of G, BP and G-BP heterojunctions. (A) Raman spectra in the range 330 to 490cm^-1^. (B) Raman spectra in the range 1100 to 2800cm^-1^.

**Figure S2.** Schematic diagram of the G-BP FET construction process. (A) empty electrode. (B) Transfer a small layer of BP into the channel. (C) Transfer PMMA/ graphene to the channel. (D) Remove PMMA from the graphene surface.

**Figure S3.** Construct optical image of G-BP FET. (A) empty electrode. (B) Transfer a small layer of BP into the channel. (C) Transfer PMMA/ graphene to the channel. (D) Remove PMMA from the graphene surface.

**Figure S4.** Raman spectral evolution of G-encapsulated BP under ambient exposure for 20 days.

**Figure S5.** Physical diagram of G-BP FET. The size is about 1×1cm^2^.

**Figure S6.** Optical image of G-BP FET. The sensor size is about 100 × 40 μm^2^(L×W).

**Figure S7.** Output characteristic curve of G-BP FET. The measuring range is 0~0.1V. *V*g The value ranges from 0 to 0.4V, and the step is 0.1V. When *V*d is fixed, the *I*d increases with the increase of *V*g, which conforms to the characteristics of p-type semiconductors. The linear characteristic of the output characteristic curve indicates that the device has a highly stable ohmic contact.

**Figure S8.** Schematic diagram of EDL.

**Figure S9.** Effect of light on the channel current of pristine graphene FET. After the addition of light, the channel current exhibited minimal change, highlighting the necessity of BP in the G-BP FET.

**Figure S10.** Transfer characteristic curves of G-BP FET under different test conditions. In the dark, the addition of Aβ_42_ resulted in a Δ*V*_Dirac_ of 5 mV. However, exposure to light significantly increased this value to 20 mV.

**Figure S11.** Response of pristine graphene FET to 100pM Aβ_42_.

**Figure S12.** Stability of G-BP FET assay system with on-off illumination for 9 times.

**Figure S13.** The light response curves of 100 pM Aβ_42_ were detected by the same G-BP FET biosensor on the 1st and 20th days.

**Figure S14.** Detection of Aβ_42_ biomarkers in serum. (A) Schematic diagram of detecting Aβ_42_ by G-BP FET. (B) Real-time response of G-BP FET to Aβ_42_ in serum in dark state. (C) The response curve of G-BP FET biosensor changed with time after adding Aβ_42_ with concentration of 0~100 pM to serum. From 10s onwards, the device is exposed to light. (D) Relationship between Aβ_42_ concentration and response signal. Where the intersection of noise level and curve is LOD. LOD is 4.440 aM in the dark and 1.932 aM in the light.

**Figure S15.** The variation trend of drain current over time during the antibody modification process.
